# Multifunctional Chitosan/Mn(II) Complexes: Preparation, Catalytic Activity in Imine Synthesis and Aldol Reaction, and Effect on Milk Fermentation/Post-Acidification

**DOI:** 10.3390/molecules30234522

**Published:** 2025-11-23

**Authors:** Roman A. Golubev, Andrey A. Nikolaev, Daria I. Semenkova, Anton R. Egorov, Linh V. Nguyen, Rovshan H. Nazarov, Anatoly A. Kirichuk, Vasili V. Rubanik, Tatsiana V. Shakola, Irina S. Garkushina, Wanjun Liu, Alexander G. Tskhovrebov, Andreii S. Kritchenkov

**Affiliations:** 1 Department of Human Ecology and Bioelementology, RUDN University, 6 Miklukho-Maklaya St., Moscow 117198, Russia; asdfdss.asdasf@yandex.ru (R.A.G.); andreynikolaev2001@yandex.ru (A.A.N.); darya.semenkova02@mail.ru (D.I.S.); sab.icex@mail.ru (A.R.E.); linhno2109@gmail.com (L.V.N.); kirichuk-aa@rudn.ru (A.A.K.); bychkovskaya-t@mail.ru (T.V.S.); alexander.tskhovrebov@gmail.com (A.G.T.); 2 Institute of Technical Acoustics NAS of Belarus, Ludnikova Prosp. 13, 210009 Vitebsk, Belarus; v.v.rubanik@tut.by; 3 Institute of Chemistry of Additives, Beyukshor Highway, Block 2062, Baku AZ 1029, Azerbaijan; rovsenhafiz@mail.ru; 4 NRC «Kurchatov Institute»-PNPI-IMC, Bolshoi VO 31, St. Petersburg 199004, Russia; irin-g16@yandex.ru; 5 Shanghai Frontiers Science Center of Advanced Textiles, College of Textiles, Donghua University, Shanghai 201620, China; wjliuok@dhu.edu.cn

**Keywords:** chitosan, manganese(II), nanoparticles, catalysis, oxidative coupling of benzylamine, aldol reaction, milk fermentation

## Abstract

Herein, we prepared nanoparticles of chitosan–manganese(II) complexes in different molar ratios (1:2, 1:1, and 2:1) and fully characterized them using dynamic and electrophoretic light scattering, X-ray diffraction, SEM, FTIR, and thermal analysis. Nanoparticles *Chitosan + Mn^2+^ (1:1)* have a high catalytic activity in the oxidative coupling of benzylamine, resulting in the imine formation and also in selective aldol reaction. *Chitosan + Mn^2+^ (1:1)* catalyze the reactions in the greenest solvents: water and water/ethanol mixture. Moreover, *Chitosan + Mn^2+^ (1:1)* is very easy to prepare and convenient to use. The catalyst is separated from the reaction mixture by a simple nanoporous filter or centrifugation and does not lose catalytic activity after at least ten uses. The chitosan–manganese(II) complexes reduce the milk fermentation time, demonstrating the effectiveness in accelerating the fermentation process by *Streptococcus thermophilus*. They also contribute to increasing the shelf life of fermented milk products by inhibiting the undesirable post-acidification process. We found that the optimal ratio of chitosan and Mn^2+^ to manifest the apogee of the desired effects (acceleration of milk fermentation and increase in the shelf life of the fermented product) is 1:2.

## 1. Introduction

Chitosan is one of the most common natural polymers (second only to cellulose). The key advantages of chitosan over other biopolymers include its biocompatibility, biodegradability, non-toxicity, and simplicity of its chemical modification due to the presence of a primary amino group in the macromolecule [1]. Chitosan can be successfully used in medicine, pharmaceuticals, cosmetics, agriculture, food industry, ecology, and other fields, and these findings have been thoroughly summarized in recent reviews and books [2,3,4,5,6,7]. On the other hand, manganese(II) is a very attractive metal center that is an important microelement for almost all living organisms [8]. Coordination compounds of manganese(II) are promising for use in medicinal chemistry [9], clinical diagnostics [10], catalysis (alkylation, oxidation, hydrosilylation, and CO_2_ reduction) [11], and photochemical end electronic devices, including OLEDs [12,13].

Mixing manganese(II) and chitosan inevitably leads to the formation of coordination compounds since chitosan is a chelating macromolecular ligand that is prone to complexation of d-metal cations [14]. The vast majority of the literature data on chitosan/manganese(II) complexes describes the sorption of manganese(II) by chitosan or its derivative-based sorbents [15,16]. Such complexes suffer from insufficient characterization.

Significantly fewer (but very interesting) studies have focused on the targeted synthesis of chitosan/manganese(II) complexes and their attractive applications in various fields. In view of our general interest in the application of metal complexes (*i*) in catalysis of organic transformations and (*ii*) in dairy biotechnological processes (stimulation of fermentation and inhibition of post-acidification), we found the following aspects in the literature regarding chitosan/manganese(II) complexes. First, *regarding catalysis*, chitosan/manganese(II)-based systems are attractive for oxidation of methyl phenyl sulfide [17], ethyl benzene oxidation [18,19], enzymatic catalysis [20], and dies oxidation [21]. However, in all cases, chitosan derivatives or complex Mn(II) compounds on a chitosan matrix are used. This, in turn, makes such studies less attractive in terms of simplicity and elegance of synthesis. Second, *regarding dairy biotechnological processes*, the literature contains examples of the use of chitosan for stimulating fermentation [22] and inhibition of the undesirable process of post-acidification [23,24]. However, the literature currently lacks an evaluation of the effects of manganese salts and chitosan/manganese(II) complex on both fermentation and post-acidification processes.

To fill these gaps, we set out to prepare and characterize chitosan and manganese(II) complexes in various molar ratios (1:2, 1:1, and 2:1). In initial experiments, we first found that these complexes formed as nanoparticles. In view of our interest in catalytic reactions, we studied the catalytic activity of the complexes in oxidative coupling of benzylamine and in aldol reaction. Based on our experience and curiosity in dairy biotechnological processes, we evaluated the effect of chitosan and manganese(II) complexes on both milk fermentation and post-acidification processes. Thus, this work consists of a predominantly chemical part (synthesis, characterization, and study of the catalytic properties of chitosan/manganese(II) complexes) and a biological part (study of effects of the complexes on milk fermentation and post-acidification). The results of these studies and their detailed discussion are presented in the sections that follow.

## 2. Results and Discussion

### 2.1. Chemistry

#### 2.1.1. Synthesis of Complexes

For synthesis of chitosan/manganese complexes, we used high molecular weight chitosan (200 kDa). Since high molecular weight chitosans are insoluble in water, we used 1% acetic acid as a solvent for the synthetic procedures. As a source of the metal center (Mn^2+^ ions), we chose highly water-soluble manganese(II) chloride tetrahydrate. A polymer/Mn^2+^ ratio was 1:2, 1:1, or 2:1 mono-mol/mol.

**Figure 1:**
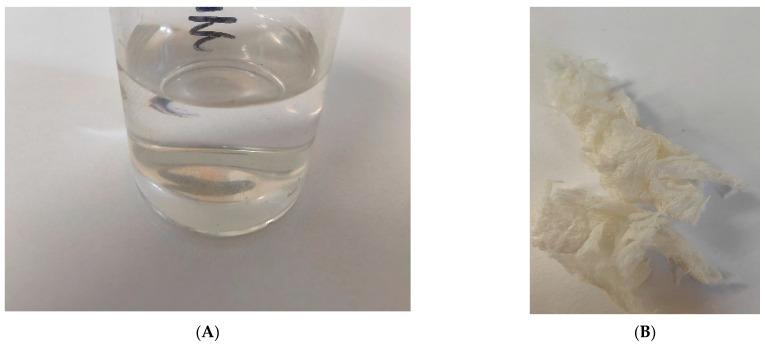
Suspension (**A**) and pale, porous, cotton-like freeze-dried *Chitosan + Mn^2+^ (1:1)* complex (**B**).

All resultant chitosan-based complexes (named *Chitosan + Mn^2+^ (1:2)*, *Chitosan + Mn^2+^ (1:1)*, and *Chitosan + Mn^2+^ (2:1)*) provided transparent suspensions (Figure 1A). After freeze-drying, the complexes appeared as fine pale, porous, cotton-like materials (Figure 1B). The synthesized complexes are easily redispersed in water after freeze-drying. The resultant suspensions are stable for a month and then undergo gradual coagulation.

ICP analysis confirmed quantitative content of manganese in the samples. Thus, the percentage in the samples *Chitosan + Mn^2+^ (1:2)*, *Chitosan + Mn^2+^ (1:1)*, and *Chitosan + Mn^2+^ (2:1)*, respectively, is as follows: 32.18%, 21.36%, and 12.52%.

#### 2.1.2. Dynamic and Electrophoretic Light Scattering Studies

Stability of the suspensions is largely determined by the size of dispersed microparticles and their zeta potential [25]. Generally, a decrease in hydrodynamic diameter of microparticles results in an increase in stability of their suspensions [26]. Zeta potential is an extremely important indicator of the stability of microparticles in a liquid medium. A high (ca. 25 mV and more) absolute value of the zeta potential determines increased stability of the colloidal system, i.e., microsuspension [27].

In this study, we evaluated the hydrodynamic diameter and zeta potential of the prepared chitosan-based complexes immediately after synthesis, after redispersion, and also during 150 days of suspension in the dark at 5 °C.

Analysis of the starting chitosan solution by dynamic light scattering expectedly showed a rapidly changing pattern of polymodal size distribution with sharply changing intensities and peak positions. This fact is not surprising and is explained by changes in sizes of macromolecular coils as a result of thermal motion due to pronounced segmental mobility, as well as molecular-mass and compositional heterogeneity of chitosan [28].

**Figure 2:**
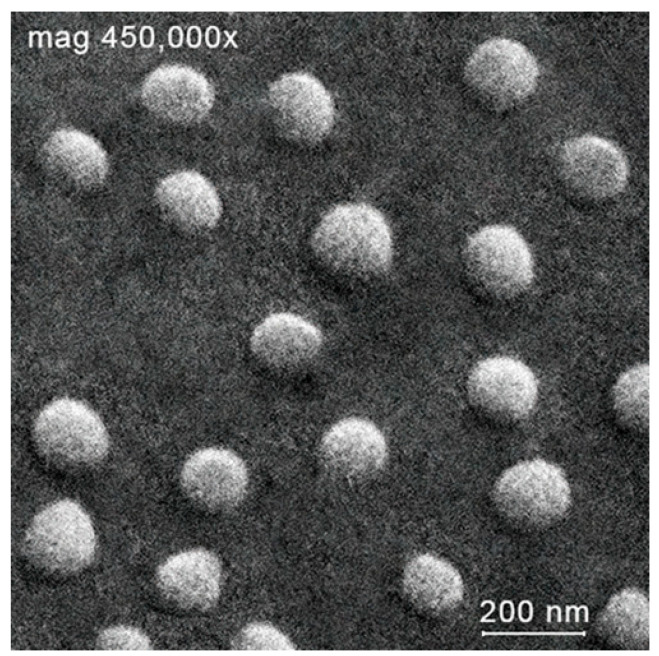
Scanning electron microscopy of microparticles *Chitosan + Mn^2+^ (1:2)*.

Almost immediately after mixing the chitosan and manganese(II) salt solutions, the situation changed dramatically; we detected the unimodal size distribution with constancy of intensity and peak position. These changes indicate formation of chitosan–metal complexes. Thus, a few minutes after starting mixing chitosan and manganese chloride solutions, the reaction mixtures gave rise to microparticles with a hydrodynamic diameter of ca. 150 nm for *Chitosan + Mn^2+^ 1:2*, ca. 274 nm for *Chitosan + Mn^2+^ 1:1*, and ca. 174 nm *for Chitosan + Mn^2+^ 2:1* with unimodal size distribution. Scanning electron microscopy (see example in Figure 2) confirms the unimodal distribution and also shows the spherical shape of the resultant microparticles. All formed microparticles have a fairly high positive zeta potential value. (ca. 20 mV *for Chitosan + Mn^2+^ (1:2)*, ca. 30 mV for *Chitosan + Mn^2+^ (1:1)*, and ca. 20 mV *for Chitosan + Mn^2+^ (2:1)*). After lyophilization, the prepared microparticles completely recover their size, shape, and zeta potential within 30 min, and this indicates their high redispersibility.

**Figure 3:**
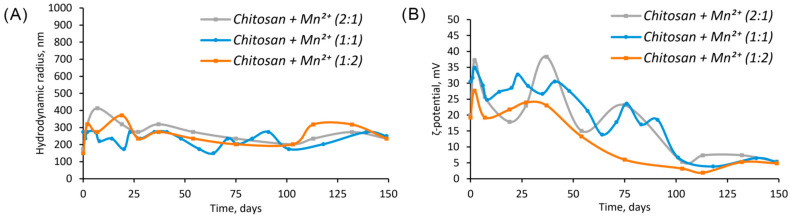
Hydrodynamic radii (**A**) and ζ-potentials (**B**) of the *chitosan–Mn^2+^ systems* (the relative standard deviation for all points was below 5%).

The hydrodynamic radii are a relatively constant value and fluctuated around 273 ± 64 nm (*Chitosan + Mn^2+^ (2:1)*), 233 ± 41 nm (*Chitosan + Mn^2+^ (1:1)*) and 261 ± 63 nm (*Chitosan + Mn^2+^ (1:2)*) during the experiment (150 days, Figure 3A).

Zeta potential of microparticles is more subject to fluctuations than their sizes. The zeta potential of all microparticles of the obtained complexes had a tendency to change over time from 37 mV (2 day) to 5 mV (150 day) for *Chitosan + Mn^2+^ 2:1*, from 35 mV (2 day) to 5 mV (150 day) for *Chitosan + Mn^2+^ 1:1*, and from 37 mV (2 day) to 5 mV (150 day) for *Chitosan + Mn^2+^ 2:1* (Figure 3B).

A month after the start of the experiment, we observed gradual weak turbidity and coagulation as a result of monotonous destabilization of the complexes due to a decrease in the aggregate stability of the particles. Aggregate stability, in turn, decreases due to a decrease in the absolute value of the ζ-potential (Figure 3B).

We summarized the statistical and correlation analysis data for the components “hydrodynamic radius–time”, “ζ-potential–time” and “hydrodynamic radius–ζ-potential” in Table 1 over 150 days of experiment.

Statistical and correlation analysis of experimental data showed that there is no relationship between the parameters “hydrodynamic radius” and “time”, as well as “hydrodynamic radius” and “ζ-potential” for the microparticles of the synthesized complexes since the *p*-value is unreliable (*p* < 0.05) and poorly describes the nature of the relationship between these quantities. In addition, the correlation coefficients have a low value (r < 0.65). Statistically unreliable values of the correlation coefficient (crossed out in the table) at *p* < 0.05 for the obtained systems mean the absence of any statistically significant relationship between the parameters, which in turn means the impossibility of further mathematical processing and functional approximation.

On the contrary, there is a clear statistical dependence between the parameters “ζ-potential” and “time”. All microparticles of the synthesized complexes are described by a medium-strong inverse dependence, which means a statistically reliable decrease in the value of the ζ-potential over time. A strong correlation dependence (r = ±0.66–±0.99) presupposes the possibility of approximating the value of the ζ-potential from time by a mathematical function.

The obtained results indicate that the synthesized systems are not equilibrium. This can be explained by rearrangements in the polymer chain of chitosan, for example, processes of gradual depolymerization of the macromolecule, reactions of rare cross-linking, partial oxidative degradation of chitosan, etc. The manganese(II) center, in turn, can also affect the stability of polymer–metal systems (competitive interaction of solvent molecules with the metal center, and change in the reactivity of the ligand [29]).

We consider the obtained systems as potential extenders of the shelf life of products based on fermented milk (e.g., yoghurts). The conventional shelf life of such products is 7–10 days. The microparticles of the synthesized complexes are stable for at least a month (see Figure 3); therefore, they are suitable for the chosen purpose. However, since the complexes are nonequilibrium systems, they should be produced as a lyophilizate and redispersed immediately before use.

#### 2.1.3. FTIR Analysis

**Figure 4:**
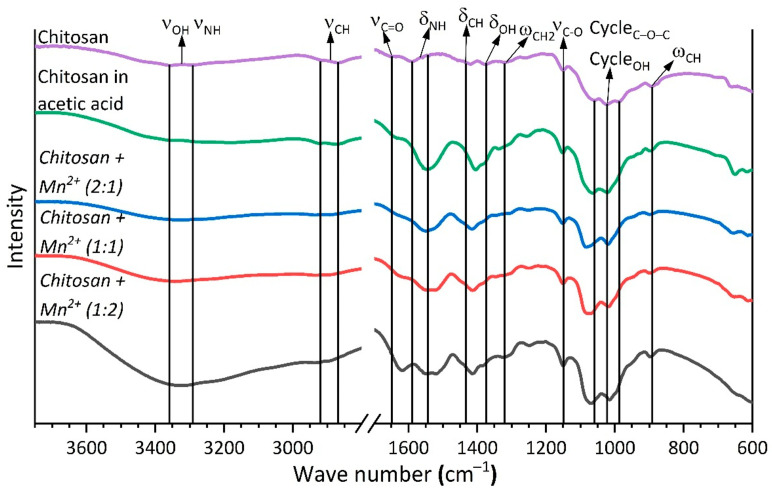
IR spectrum of chitosan and complexes with Mn^2+^.

We also used FTIR spectroscopy to further investigate the interactions between chitosan and the manganese(II) center in the prepared complexes. We used a set of previously published data [29,30,31] to identify the absorption bands in the obtained spectra (Figure 4). The wave numbers of the absorption maxima are presented in Table 2.

The coordination of chitosan to the manganese(II) center does not cause any noticeable changes in the positions of the characteristic bands due to vibrations of the C–O–C bonds of the pyranose ring and the ring-bound O–H groups (∆ 10 cm^−1^). However, the interaction of chitosan with Mn^2+^ results in a noticeable shift of the C=O group vibration band (∆ 30 cm^−1^) and the vibration bands of the N–H and C–H bonds, and also O–H bonds not bound directly to the pyranose ring (∆ 5–15 cm^−1^ for deformation vibrations, ∆ 50 cm^−1^ for stretching vibrations of the N–H, O–H bonds, and ∆ 20 cm^−1^ for stretching vibrations of the C–H bonds). These results indicate the coordination of chitosan to the manganese(II) center through the C=O, N–H, and not bound directly to the pyranose ring O–H functional groups [32,33,34].

#### 2.1.4. X-Ray Diffraction Studies

**Figure 5:**
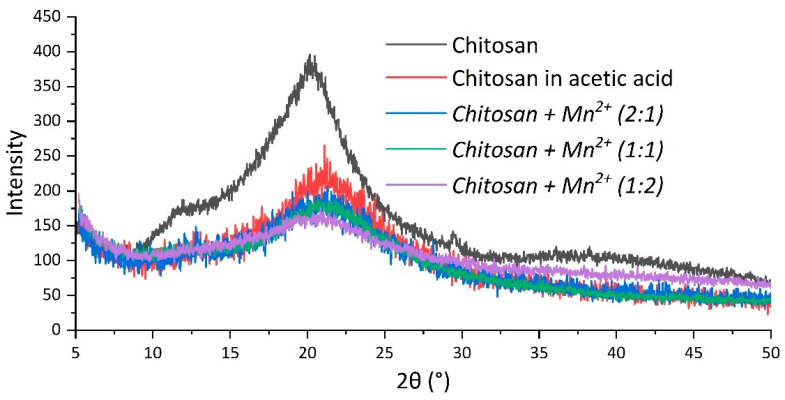
Diffractograms of chitosan and chitosan complexes with Mn^2+^.

The obtained complexes were also characterized by X-ray structural analysis (powder diffraction). The diffraction patterns (Figure 5) demonstrate that the synthesized complexes, as well as the starting chitosan, are X-ray amorphous. However, the intensity of the main broadened peak of chitosan is significantly higher than that of chitosan in acetic acid (chitosan acetate). This fact indirectly, at a qualitative level, indicates a significantly higher amorphism of chitosan acetate than that of the starting chitosan. Thus, protonation of chitosan with acetic acid leads to a very noticeable increase in amorphism. The introduction of a Mn(II) center into the protonated chitosan macromolecule also leads to a further slight increase in amorphism, although this effect is significantly less pronounced than the effect of protonation. This is evidenced by a monotonic decrease in the intensity of the peak centered at ca. 20–22° 2θ in the series *Chitosan + Mn^2+^ (2:1)* − *Chitosan + Mn^2+^ (1:1) − Chitosan + Mn^2+^ (2:2)* (Figure 5).

The diffraction patterns also show a change in the peak profile of chitosan in the sample after dissolution in acetic acid compared to the starting polymer. We observed a shift in the main peak of chitosan, and this indicates a rearrangement of the polymer structure because of the formation of chitosan acetate [35,36]. After the interaction of chitosan with manganese(II) chloride, we did not find any peaks in the diffraction patterns of the obtained complexes corresponding to the original salt.

#### 2.1.5. Differential Thermal and Thermogravimetric Analysis

**Figure 6:**
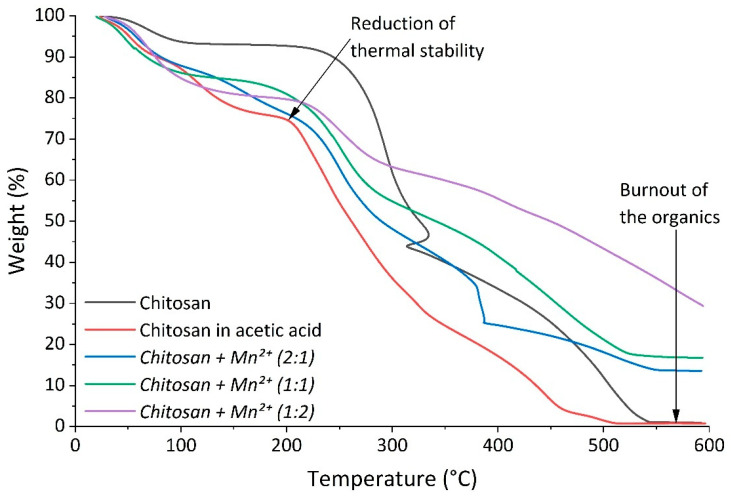
TGA curves of chitosan and chitosan–Mn^2+^.

**Figure 7:**
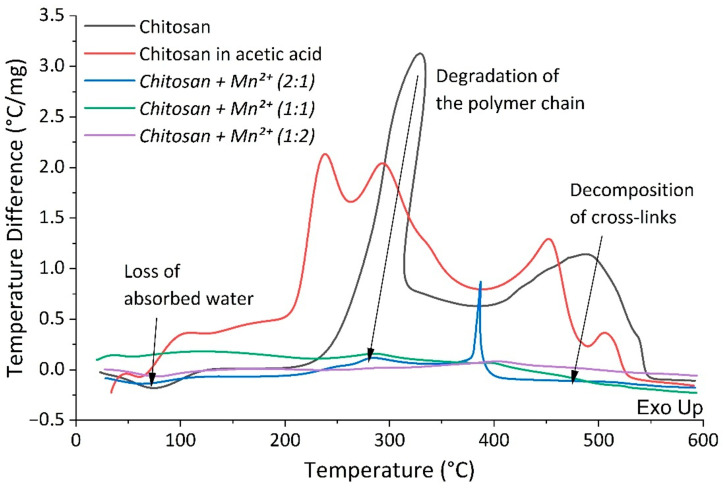
DTA curves of chitosan and chitosan–Mn^2+^.

We used differential thermal and thermogravimetric analysis in N_2_ atmosphere to study the thermal stability of the obtained sample complexes and to evaluate the effect of introducing Mn^2+^ ion into the polymer matrix. Thermal curves of the synthesized complexes (Figure 6 and Figure 7) show that the decomposition of their chitosan occurs in two main stages [37,38,39,40].

The first stage occurs at a temperature of about 60 °C and is characterized by a mass loss from 7% to 20%. This stage is accompanied by an endothermic effect due to the evaporation of water bound to the polymer matrix and/or coordinated to the manganese(II) center [41]. Table 3 demonstrates a marked increase in water content in the complexes associated with an increase in manganese content. This is not surprising since the manganese(II) center prefers to coordinate the so-called hard Lewis ligands, primarily H_2_O.

The second stage begins at ca. 225 °C and continues up to 580 °C. This stage results in mass loss of 92% for chitosan, 73.29% for *Chitosan + Mn^2+^ (2:1)*, 67.95% for *Chitosan + Mn^2+^ (1:1),* and 50.81% for *Chitosan + Mn^2+^ (1:2)*. It is associated with gradual degradation of the polymer chain and the burning of its decay products. The degradation of chitosan is manifested by the cleavage of glycosidic bonds, then the resulting oligomers decompose with the subsequent formation of acetic, butyric acids, as well as lower fatty acids [38].

#### 2.1.6. Catalytic Activity of Chitosan–Mn^2+^ Systems in Oxidative Coupling of Benzylamine

**Scheme 1:**
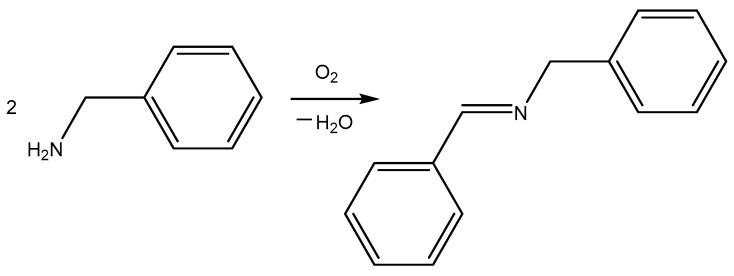
The model reaction of imine synthesis from benzylamine.

Imines are very attractive for organic chemistry because they are building blocks in the synthesis of many compounds that are important in technology, medicine, agriculture, ecology, and so on [42], that is, imines are universal molecules in organic synthesis, especially in the preparation of various heterocyclic systems [43]. Conventional protocols for the preparation of imines are based on the nucleophilic addition–elimination (*Ad_N_-E*) reaction of primary amines (acting as the nucleophilic reagent) with aldehydes (the electrophilic substrate). Yet recently, we have witnessed a steady trend toward expanding approaches to imine synthesis (dehydrogenation of secondary amines, coupling of alcohols and amines, and oxidative coupling of amines). Most of these processes require catalysts that are typically used in metal complex catalysis [42]. The development of these approaches results in new, simple, and preparatively convenient routes to imines and significantly improves the yields of the target products and their isolation procedure. In this study, we used chitosan–Mn^2+^ systems as catalysts for model reaction of imine synthesis from benzylamine (Scheme 1).

We evaluated the catalytic properties of the elaborated nanoparticles under the following reaction conditions: heating the reaction mixture for 48 h at 80 °C in the presence of 3 mol% (based on Mn) of the tested catalyst. We first decided to evaluate the catalytic effect of the prepared Chitosan–Mn^2+^ systems in the greenest solvent, i.e., water (Table 4, Entries 1–3). However, we achieved only low yields (about 20%). We decided to lower the solvent polarity and used methanol (Table 4, Entries 4–6). In this case, the stating materials remained intact, and we observed only traces of the desired product. Thus, the low yields in aqueous media are not due to the low solubility of benzylamine in water, as benzylamine is soluble in methanol, but the yield of the target imine in methanol is lower than in water. Furthermore, we attempted to use 10% trimethylbenzylammonium chloride as a phase-transfer catalyst to improve the yield of the heterogeneous reaction in water. However, this attempt did not result in any noticeable increase in the imine yield. We believe that the low yields in water and, especially, methanol are due to the *O-*coordination of these solvents to the Mn(II) center, which can lead to deactivation of the so-called catalytically active sites. The catalytic reaction in toluene afforded slightly higher yields (27–34%). Thus, switching to an aromatic solvent increased the conversion of the starting amine to the target imine (Table 4, Entries 7–9). Significant success was achieved when we completely eliminated the solvent (Table 4, Entries 10–12). The solvent-free catalytic reaction resulted in high imine yields (53–80%). Solvent-free transformations, along with reactions in the greenest solvent (water) are of paramount importance in so-called green chemistry [44].

In all entries, we observed that *Chitosan + Mn^2+^ (1:1)* exhibited the greatest catalytic effect. We believe that this may be due to the smaller size of the nanoparticles (see Table 1). Furthermore, the increase in reaction yields demonstrated a dramatic difference in the catalytic effect of the tested nanoparticles (*Chitosan + Mn^2+^ (2:1)*, *Chitosan + Mn^2+^ (1:1)*, and *Chitosan + Mn^2+^ (1:2)*). *Chitosan + Mn^2+^ (1:1)* nanoparticles exhibited the highest catalytic activity.

However, even the solvent-free protocol and the *Chitosan + Mn^2+^ (1:1)* catalytic system did not provide a 100% imine yield. This prompted us to increase the catalyst content to 2 mol% (Table 4, Entry 14). As expected, this increase in catalyst concentration to 2 mol% resulted in a higher product yield (93%). Finally, 3 mol% of *Chitosan + Mn^2+^ (1:1)* enabled complete conversion of the starting benzylamine to the target imine (Table 4, Entry 14).

The tested catalytic reaction does not proceed under anaerobic conditions. Thus, oxygen is essential, as it obviously functions as an oxidizing agent in the organic transformation.

We also performed a blank experiment, i.e., without any catalyst (Table 4, Entry 13), and detected a very low (10%) yield of imine. To evaluate the necessity of combining Mn^2+^ and chitosan for the functioning of the integrated catalytic ensemble, we used 1 mol% of manganese(II) acetate in its tetrahydrate form (Table 4, Entry 16) or chitosan (Table 4, Entry 17). Using manganese(II) acetate, we achieved a yield of 15%, and this is only slightly higher than under catalyst-free conditions. The use of chitosan, on the contrary, reduces the imine yield even compared to the complete absence of catalyst (6%). Thus, pure chitosan without any Mn(II) center inhibits the oxidative coupling, and only the complex of chitosan and Mn^2+^ (1:1) provides the highest yields.

It is also important that the catalyst can be separated from the reaction mixture by simple filtration or centrifugation. After centrifugation, *Chitosan + Mn^2+^ (1:1)* can be freeze-dried and reused for catalysis at least 10 times without loss or reduction in catalytic effect.

#### 2.1.7. Catalytic Activity of Chitosan–Mn^2+^ Systems in Aldol Reaction

Aldol reaction is one of the simplest and the oldest tools for carbon(sp^3^)–carbon(sp^3^) bond formation. The reaction includes the coupling of two carbonyl compounds. One of them contains an active methylene component (α-CH acid reaction center) and acts as a nucleophile, attacking the electrophilic carbon atom of the carbonyl group of the second carbonyl compound. Thus, the reaction affords β-hydroxycarbonyl compound [45]. Classic aldol reaction protocols require the use of strong bases (usually sodium and potassium alkoxides), non-aqueous toxic solvents (toluene and xylene), and prolonged exposure to high temperatures. This, in turn, results in dramatic drawbacks (environmental hazards, and side reactions, such as the elimination of water to form α,β-unsaturated compounds, followed by oxidation and polymerization) [46]. These complications have led to new protocols that utilize improved catalytic systems and mild conditions [47]. Most of these metal-based catalysts, although offering high selectivity and mild conditions, are sophisticated and their synthesis requires laborious methods. Here, we propose a new, efficient aldol reaction catalyst, which is prepared by simply mixing chitosan and a manganese(II) salt.

**Scheme 2:**
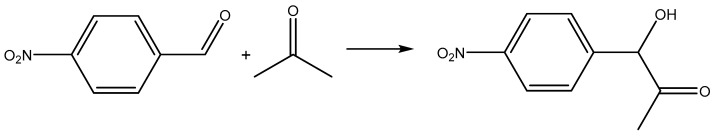
The model aldol reaction.

To evaluate the catalytic effect of the synthesized Chitosan–Mn^2+^ systems, we turned to the classical model aldol reaction of coupling 4-nitrobenzaldehyde with acetone (Scheme 2).

To study the catalytic activity of the tested catalysts for coupling 4-nitrobenzaldehyde aldehyde with acetone, we attempted to use water, the greenest solvent. Furthermore, we intended to avoid heating the reaction mixture and conducted experiments at room temperature (ca. 20 °C). We used 2 mol% of the catalysts (based on Mn), and the reaction was monitored by thick-layer chromatography (TLC) for 1 h (60 min). We considered the reaction complete when the TLC spot of the starting aldehyde disappeared, leaving only the spot of the aldol coupling product, i.e., the corresponding β-hydroxycarbonyl compound. We also monitored the reaction with ^1^H NMR spectroscopy to quantify the conversion of the starting 4-nitrobenzaldehyde to the desired product. The results are presented in Table 5.

The model reaction at room temperature in water proceeded unsatisfactorily. A total 60 min after the start of the experiment, we detected only small amounts of the aldol (Table 5, Entries 1–3). This is apparently due to the low solubility of the starting 4-nitrobenzaldehyde in water. To increase the solubility of the aromatic aldehyde, we added a small amount of ethanol to the reaction mixture (we used a solvent system H_2_O/EtOH 5/1 (volume/volume)). It should be noted that ethanol is an environmentally friendly organic solvent [48]. The starting aldehyde is soluble in the solvent system, which significantly improves the yields of the target aldol. The use of *Chitosan + Mn^2+^ (1:1)* nanoparticles as a catalyst allows for complete conversion of the starting aldehyde to the desired product (Table 5, Entries 4–6). Moreover, *Chitosan + Mn^2+^ (1:1)* nanoparticles are also easily separated from the reaction mixture and do not lose catalytic activity, as in the case of the aldol reaction (see Section 2.1.7). Other nanoparticles proved less active. However, among *Chitosan + Mn^2+^ (2:1)* and *Chitosan + Mn^2+^ (1:2)*, the nanoparticles with a higher manganese(II) content exhibit slightly greater catalytic activity. The reaction proceeds selectively and under mild conditions. In the absence of a catalyst, the studied reaction is impossible, and we did not detect even a trace of the product (Table 5, Entry 7). We also demonstrated that combining both chitosan and Mn^2+^ is essential for the catalytic effect, since neither chitosan nor Mn^2+^ alone can catalyze the model aldol reaction (Table 5, Entries 8 and 9).

It is logical to assume that the low yields in an aqueous medium are due to the poor solubility of the starting aldehyde in water. Indeed, the use of 10% phase-transfer catalyst (trimethylbenzylammonium chloride, Table 5, Entry 10) led to a nearly fourfold increase in the aldol yield (up to 45%). However, we believe that the use of an H_2_O/EtOH solvent system is more appropriate because (i) H_2_O/EtOH is significantly more environmentally friendly than trimethylbenzylammonium chloride, (ii) all other things being equal, H_2_O/EtOH provides higher yields of the product, and (iii) H_2_O/EtOH is cheaper and more readily available.

### 2.2. Biology

Intensification of enzymatic processes, enhancing the growth and activity of desirable producing microorganisms, is a task of paramount importance in dairy biotechnology [22]. On the other hand, post-acidification is an undesirable phenomenon in fermented milk products, characterized by acidification outside the optimal pH range due to the activity of microflora [49]. This process triggers an increase in hydrophobic and electrostatic interactions between protein molecules, leading to (i) the enlargement and aggregation of casein particles and (ii) partial restructuring of the protein network within the product [50]. Consequently, post-acidification significantly reduces the shelf life of fermented dairy products. Thus, the development of systems capable of intensifying fermentation and inhibiting post-acid is of great importance for dairy biotechnology.

#### 2.2.1. Effect of Chitosan–Mn^2+^ Systems on Fermentation: Kinetics, pH Changes, and Dynamics of CFU Number Changes

At the first stage, we investigated the effect of the obtained Chitosan–Mn^2+^ systems (*Chitosan + Mn^2+^ (2:1)*, *Chitosan + Mn^2+^ (1:1)*, and *Chitosan + Mn^2+^ (1:2*)) on fermented milk pH changes and fermentation kinetics. We compared the effects of the synthesized systems with manganese chloride. We used different masses of the tested samples which contained the same amount (1.3 mg per 1000 mL of milk) of Mn^2+^ cations. We also carried out a blank experiment which excludes the use of any additives. The rate and depth of pH change dramatically depends on the type of additive used.

**Figure 8:**
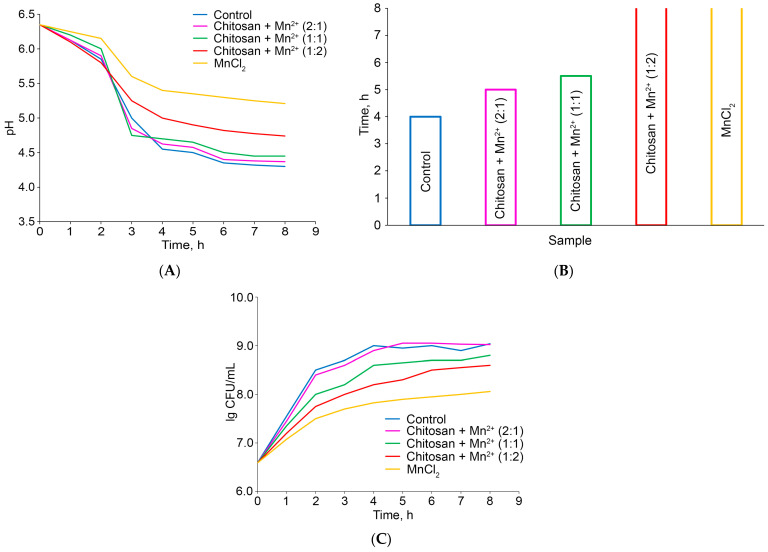
pH changes of the milk under fermentation (**A**); fermentation time (**B**); changes in the number of CFU during fermentation (**C**).

The results of the experiment are shown in Figure 8. The highest rate of pH decrease was observed in the case of the control experiment, i.e., without any additives. Eight hours after the start of the fermentation experiment, the pH value reached 4.30. Manganese(II) chloride causes the least acidification of milk in the same period of time (pH = 5.21). According to their ability to reduce the pH of fermented milk, the Chitosan–Mn^2+^ systems are arranged in the following order: *Chitosan + Mn^2+^ (1:2)* (pH after 8 h 4.74) < *Chitosan + Mn^2+^ (1:1)* (pH after 8 h 4.45) < *Chitosan + Mn^2+^ (2:1)* (pH after 8 h 4.37). Since the amount of manganese(II) is the same in all tested systems, the observed effect is related to the amount of chitosan: the greatest acidification of fermented milk is caused by *Chitosan + Mn^2+^* (*2:1)* system containing the largest amount of chitosan (Figure 8A). We found a similar pattern for the milk fermentation time. The fermentation time is considered to be when the pH reaches 4.60 [51,52]. The fermentation time was inversely proportional to the fermentation rate. The shortest fermentation time (see Figure 8B) was observed in the control experiment and in the case of using the *Chitosan + Mn^2+^ (2:1)* and *Chitosan + Mn^2+^ (1:1)* systems (4 h and 5 h, respectively). Figure 8C displays that the fermentation rate decreases with a decrease in the chitosan content in the system. For the *Chitosan + Mn^2+^ (1:2)* system and manganese chloride, the fermentation time is more than 8 h.

At the next stage, we assessed the ability of the tested additives to influence the number of colony-forming units (CFU) producing bacteria *St. thermophilus* in fermented milk in comparison with the control experiment (without any additives). The initial number of CFU in milk was 3.93 × 10^6^ CFU/mL. Over 8 h of the experiment, the maximum increase in the number of CFU was achieved in the case of the control experiment: the number of CFU/mL increased to 1.12 × 10^9^. The use of *Chitosan + Mn^2+^ 2:1* for fermentation leads to a similar result (1.04 × 10^6^ CFU/mL). At the same time, the graphical dependence of lg CFU on time under the influence of the *Chitosan + Mn^2+^ (2:1)* system is close in nature to that for the control experiment; even the analysis of variance did not reveal any significant differences between the trends of these two curves (Figure 8C). Manganese(II) chloride has a pronounced inhibitory effect on the growth of producing bacteria. Under the action of MnCl_2_, we were able to achieve an increase in the CFU/mL value only up to 1.17 × 10^8^, which is an order of magnitude less than in the case of the control experiment or *Chitosan + Mn^2+^ (2:1)* system. The other tested Chitosan–Mn^2+^ systems demonstrate an intermediate position. Their tendency to inhibit the growth of *St. thermophilus* in fermented milk increases with decreasing chitosan content. Thus, the obtained results are consistent with those for changing the pH and fermentation time: the tested additives with the highest chitosan content not only cause the greatest souring of milk with the highest fermentation rate, but also determine the highest growth rate of *St. thermophilus*. This result is consistent with the views that the fermentation kinetics are associated with the growth of *St. thermophilus* [53,54]. A separate, interesting direction arising from this work is to elucidate the mechanism by which chitosan–Mn^2+^ complexes influence bacterial growth. This effect may be either nutritional (Mn^2+^ as a micronutrient) or physicochemical. Our future work will be focused on exploring this issue.

#### 2.2.2. Effect of Chitosan–Mn^2+^ Systems on Post-Acidification: Dynamics of Changes in the Number of CFU and pH of the Fermented Product During Storage

**Figure 9:**
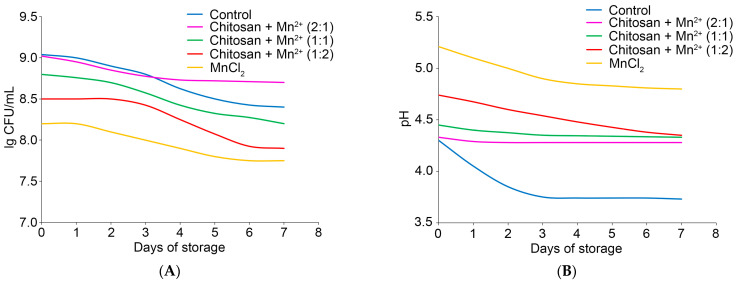
Monitoring *St. thermophilus* (**A**) and pH (**B**) levels during storage.

In the final stages of the experiment, we monitored the change in pH and the number of CFU during the storage of fermented milk for 7 days, conducting the experiment under thermostatting conditions at 25 °C. The number of CFU/mL tends to decrease during the experiment, but this trend is different for the tested samples depending on the additive (Figure 9A). In the control experiment, the CFU/mL value decreases from about 10^9^ to about 10^8.4^, demonstrating a pronounced almost linear downward trend. Under the influence of the *Chitosan + Mn^2+^ (2:1)* system, a less pronounced tendency to decrease in the number of producing bacteria is observed and the CFU value decreases only from about 10^9^ to 10^8.7^, with the main decrease observed in the first 3 days of the experiment, and subsequently the level of *St. thermophilus* remains approximately constant with a tendency only to an insignificant monotonic decrease. In the case of other additives, the level of bacteria decreases significantly, and this effect is most pronounced for the so-called “pure” manganese chloride.

The patterns of pH changes in fermented milk during storage are similar (Figure 9B). In all cases, we observed a decrease in pH despite a decrease in the number of bacteria. This effect can apparently be explained by the growth of other microorganisms that cause the so-called post-acidification of fermented milk, as well as a cascade of side biochemical reactions [52]. The complete and detailed mechanism of the undesirable process of post-acidification still remains unclear and debatable. Some researchers believe that the post-acidification in fermented milks is generally related to the lowering of pH and to the formation of bitter peptides, excluding the growth of unfavorable bacteria. Other publications indicate that undesirable continued growth of microorganisms may be the cause of post-acidification [49].

The greatest post-acidification was observed in the control experiment. This results in the product becoming unsuitable on the 4th day of storage at 25 °C, since the pH of the product reaches an undesirable value, below 4.4 [52,55,56]. The best result was achieved using the *Chitosan + Mn^2+^ (2:1)* system as an additive: the pH level was almost the same with a slight insignificant tendency to decrease.

Thus, the fermented milk retains its shelf life almost two times longer compared to the control when using the *Chitosan + Mn^2+^ (2:1)* system. It is worth noting that yoghurts are usually stored at lower temperatures. However, if we managed to achieve an extension of the shelf life even at 25 °C, then at lower temperatures, obviously, this effect will be undoubtedly more pronounced.

## 3. Materials and Methods

### 3.1. Chemicals and Solvents

Chitosan of viscosity-average molecular weight 200 kDa and degree of acetylation 8% was purchased from Bioprogress (Moscow, Russia). By the term “chitosan”, we mean the powder of the commercial chitosan used. By the term “chitosan in acetic acid”, we mean the dry lyophilizate of chitosan obtained by dissolving commercial chitosan in acetic acid, followed by purification by dialysis against distilled water from excess acid and subsequent lyophilic drying. Manganese(II) chloride tetrahydrate were from Reachim (Moscow, Russia). Other chemicals and solvents were also from commercial sources and used as received without any further purification.

### 3.2. Instrumentation

The apparent hydrodynamic radii and ζ-potential of nanoparticles in water were estimated at room temperature (about 25 °C) using a Photocor Compact-Z instrument (Moscow, Russia) at λ = 638 nm, θ = 90° for radii (10 scans, each one for 10 s), and θ = 20° for ζ-potential (three scans, each one for 60 s).

pH was measured potentiometrically using a PHS-3C device (Shanghai Precision Scientific Instrument Co., Ltd., Shanghai, China).

Inductively coupled plasma-atomic emission spectroscopy measurements (ICP) were performed at Leeman ICP-AES Prodigy XP spectrometer (Hudson, NH, USA).

IR spectroscopy was recorded on a Shimadzu IRSpirit (Nakagyo-ku, Japan) at 4000 to 400 cm^−1^.

X-ray diffraction analysis was carried out on a Dron-7 X-ray diffractometer (Guangzhou Betop Scientific Technology Co., Ltd., Guangzhou, China), using a 2θ angle interval from 5° to 50° with scanning step ∆2θ = 0.02° and exposure of 3 s per point. Cu Kα radiation (Ni filter) was used, which was subsequently decomposed into Kα_1_ and Kα_2_ components during the processing of the spectra.

Differential thermal analysis (DTA) and thermogravimetric analysis (TGA) were performed on the SDT Q600 (TA Instruments, New Castle, DE, USA) using a heating rate of 10 °C/min in the temperature range from 30 °C to 600 °C in N_2_ atmosphere.

The ^1^H NMR spectra were recorded on a Bruker Avance II spectrometer (Billerica, MA, USA) at an operating frequency of 400 MHz.

Thin-layer chromatography (TLC) was performed on Merck 60 F_254_SiO_2_ plates (Rahway, NJ, USA) with 1:1 (*v*:*v*) acetone–chloroform as the eluent.

### 3.3. Preparation of Chitosan/Manganese(II) Complexes

Chitosan (0.025 g) was dissolved in 25 mL of 1% CH_3_COOH and stirred for 30 min with a mechanical stirrer. MnCl_2_ × 4H_2_O (0.1979 g) was dissolved in 5 mL of distilled water and stirred for 30 min to produce 0.2M solution. The manganese(II) salt solution was poured as a thin stream to the vigorously stirred solution of chitosan to produce 1:2, 1:1, or 2:1 mono-mol/mol polymer/Mn^2+^ ratio solutions, and the resulting mixtures was intensely stirred for 5 min (mono-mol is a structural unit in the polymer chain of chitosan–glucosamine or acetylglucosamine unit). The resultant suspensions were frozen and freeze-dried.

### 3.4. Catalytic Synthesis of Imine by Oxidative Coupling of Benzylamine

The reaction flask was charged with benzylamine (0.050 mol), solvent (30 mL, see Table 4: H_2_O, MeOH, or MePh), internal standard hexamethyl disiloxane (0.010 mol), the tested catalyst (see Table 4: 1, 2, or 3 mol% based on Mn; the content of Mn in the chitosan/manganese(II) complexes tested as catalysts is described in Section 2.1). The reaction mixture was stirred during 48 h under air at 80 °C. The solvent was evaporated under vacuum. The aliquot 10.0 mg was dissolved in CDCl_3_ and analyzed by ^1^H NMR spectroscopy. The conversion of benzylamine into the corresponding imine was calculated as 100 × I(H from CH=N of the imine), while I(H of hexamethyl disiloxane) = 18.

### 3.5. Catalytic Aldol Reaction

The reaction vial was charged by 4-nitrobenzaldehyde (0.010 mol), acetone (0.030 mol), catalyst (see Table 5; 3 mol% based on Mn; the content of Mn in the chitosan/manganese(II) complexes tested as catalysts is described in Section 2.1) and solvent (30 mL, H_2_O, or H_2_O/EtOH 5/1, *v*/*v*). The reaction mixture was stirred at room temperature for 5, 15, 30, or 60 min. The catalyst was immediately removed by filtration. The solvent was evaporated under vacuum and the solid residue was quantitatively analyzed by ^1^H NMR spectroscopy in DMSO-d_6_ solution. The conversion of 4-nitrobenzaldehyde into the aldol was calculated as 100/(I(H^1^) + 1), while I(H^2^) = 1 (where I(H^1^) is the integral intensity of the aldehyde group proton of *p-*nitrobenzaldehyde, and I(H^2^) is integral intensity of α-CH proton the aldol).

### 3.6. Fermentation Study

Milk purchased from Neva-Milk Company (Saint Petersburg, Russia) was used for experiments. A total of 1000 mL of the milk was poured into a flat-bottomed flask, and 4.64 mg MnCl_2_ × 4H_2_O or 12.30 mg *Chitosan + Mn^2+^ (1:2)* or 8.12 mg *Chitosan + Mn^2+^ (1:1)* or 6.13 mg *Chitosan + Mn^2+^ (2:1)* was added to the milk and stirred over 20 min under room temperature. Thus, we added different amounts of the tested samples which contain the same mass of Mn^2+^, i.e.*,* 1.30 mg. The flasks were charged by *Streptococcus thermophilus* (*St. thermophilus*, 20 g per 1000 mL of the milk) and treated for 8 h at 42 °C for fermentation. For pH monitoring, 50 mL of milk was taken at 2, 3, 4, 5, 6, 7, and 8 h of the fermentation experiment. When the pH value decreased to 4.60, fermentation was considered complete. The remaining fermented milk was immediately cooled to stop the fermentation process and was used in further experiments.

### 3.7. Post-Acidification Study

For post-acidification assessment, the cooled fermented milk was kept in plastic sterile plastic yogurt containers (1 portion was 85 mL) at 25 °C. pH and the number of colony-forming units (CFU) of *St. thermophilus* monitoring was conducted every 24 h for one week. For determination of CFU, 25 mL of the fermented milk was poured into 200 mL of 0.15% peptone water. Tenfold dilutions were used for inoculation on a nutrient medium (tryptone, sucrose, yeast extract, and potassium hydrophosphate, pH = 6.8). Colonies were counted after incubation of the inoculations at 37 °C for 24 h.

### 3.8. Statistics

The statistical significance of differences between the samples was determined by a one-way analysis of variance (ANOVA) using JMP 5.0.1 software (SAS Campus Drive, Cary, NC, USA). Mean values, where appropriate, were compared by applying the Student’s *t*-test at a significance level *p* < 0.05.

## 4. Conclusions

The results of this work can be considered from the following few perspectives.

(1) *Development and Characterization of Chitosan/Mn(II) Nanoparticles:*

- We successfully synthesized and fully characterized nanoparticles based on chitosan and manganese(II) ions in various molar ratios: *Chitosan + Mn^2+^ (1:2)*, *Chitosan + Mn^2+^ (1:1)*, and *Chitosan + Mn^2+^ (2:1);*
- These nanoparticles exhibited microscale sizes and a progressively decreasing zeta potential in aqueous medium, indicating sedimentation instability;
- ICP analysis verified the quantitative manganese content, while IR spectroscopy demonstrated the coordination of manganese(II) to the chitosan matrix.

(2) *Catalytic Activity of Chitosan/Mn(II) Nanoparticles:*

- Nanoparticles *Chitosan + Mn^2+^ (1:1)* high catalytic activity in oxidative coupling of benzylamine resulting the imine formation. The reaction proceeds selectively under green solvent-free conditions and affords quantitative yields of the product;
- Moreover, *Chitosan + Mn^2+^ (1:1)* nanoparticles are efficient catalysts for selective aldol reaction at room temperature in the greenest solvent system H_2_O/EtOH 5/1 (volume/volume);
- Catalyst *Chitosan + Mn^2+^ (1:1)* is very easy to prepare and convenient to use. The catalyst is separated from the reaction mixture by nanoporous filter or centrifugation and does not lose catalytic activity after at least ten uses.

(3) *Stimulation of Milk Fermentation and Shelf-Life Assessment of Fermented Milk Products:*

- *Chitosan + Mn^2+^ (2:1)* complex significantly reduced the milk fermentation time, demonstrating its efficiency in promoting faster fermentation;
- Over a 7-day storage period, the *Chitosan + Mn^2+^ (2:1)* system exhibited the least pronounced decline in colony-forming units (CFUs) of *Streptococcus thermophilus;*
- The pH of the fermented milk products treated with this system remained nearly constant, with only a slight decrease, indicating enhanced stability;
- Using the *Chitosan + Mn^2+^ (2:1)* system nearly doubled the shelf life of fermented milk products compared to the control.

Finally, the synthesized nanoparticles are promising candidates for further chemical and biological studies. In addition, the elaborated nanoparticles are of interest for reinforcement chitosan films with their subsequent treatment with cold plasma to prepare new film materials with attractive physicochemical and biological properties. Both these projects are underway in our group.

## Figures and Tables

**Table 1:** Statistical analysis of the characteristics of chitosan–metal systems.

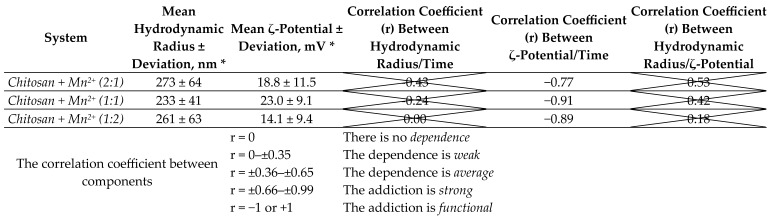

* Mean value ± SD, n = 3.

**Table 2:** Wave numbers of maxima (cm^−1^) of absorption bands of chitosan complexes with Mn^2+^.

	Chitosan	Chitosan in Acetic Acid	Chitosan + Mn^2+^ (2:1)	Chitosan + Mn^2+^ (1:1)	Chitosan + Mn^2+^ (1:2)
ω_C-H_	893	893	899	899	897
Cycle _C–O–C_ Cycle _–OH_	985, 1024, 1059	988, 1023, 1065	995, 1020, 1082	994, 1019, 1070	995, 1016, 1070
ν_C–O_	1150	1151	1152	1150	1150
ω_CH2_	1320	1302, 1338	1304, 1344	1304, 1326	1299, 1324
δ_O–H_	1375	1378	1379	1384	1383
δ_C–H_	1419, 1454	1404	1415, 1451	1413, 1451	1413, 1453
δ_N–H_	1542, 1589	1548	1549	1545, 1579	1548
ν_C=O_	1649	1634	1624	1617	1616
ν_C–H_	2868, 2914	2877, 2924	2890, 2934	2894, 2934	2894, 2936
ν_O–H_ + ν_N–H_	3291, 3352	3185, 3267, 3356	3331	3218, 3343	3212, 3325

**Table 3:** Thermal parameters of chitosan complexes with Mn^2+^.

№	Sample	Endoeffect (°C)—Loss of Absorbed Water	Weight Loss (%) of Absorbed Water	Exoeffect (°C)—Thermal Degradation of the Polymer Chain, Cleavage of Glycosidic Bonds	Exoeffect (°C)—Decomposition of Chitosan Crosslinks	Weight Loss (%) of Thermal Decomposition	Total Mass Loss (%)
1	Chitosan	76	7.09	329	489	91.79	98.88
1′	Chitosan in acetic acid	62	7.00	238, 294	453, 507	92.31	99.31
2	*Chitosan + Mn^2+^ (2:1)*	65	13.14	284	387, 513	73.29	86.43
3	*Chitosan + Mn^2+^ (1:1)*	54	15.15	286	396, 420, 501	67.95	83.10
4	*Chitosan + Mn^2+^ (1:2)*	78	19.75	285	403, 479	50.81	70.56

**Table 4:** Catalytic activity of chitosan–Mn^2+^ systems in oxidative coupling of benzylamine *.

Entry	Catalyst	Solvent	mol% of Catalyst (Based on Mn)	Yield, % **
1	*Chitosan + Mn^2+^ (2:1)*	H_2_O	1	20
2	*Chitosan + Mn^2+^ (1:1)*	H_2_O	1	22
3	*Chitosan + Mn^2+^ (1:2*)	H_2_O	1	20
4	*Chitosan + Mn^2+^ (2:1)*	MeOH	1	traces
5	*Chitosan + Mn^2+^ (1:1)*	MeOH	1	traces
6	*Chitosan + Mn^2+^ (1:2*)	MeOH	1	traces
7	*Chitosan + Mn^2+^ (2:1)*	MePh	1	27
8	*Chitosan + Mn^2+^ (1:1)*	MePh	1	34
9	*Chitosan + Mn^2+^ (1:2*)	MePh	1	25
10	*Chitosan + Mn^2+^ (2:1)*	–	1	53
11	*Chitosan + Mn^2+^ (1:1)*	–	1	80
12	*Chitosan + Mn^2+^ (1:2*)	–	1	63
13	–	–	–	10
14	*Chitosan + Mn^2+^ (1:1)*	–	2	93
15	*Chitosan + Mn^2+^ (1:1)*	–	3	100
16	*Mn^2+^*	–	1	15
17	*Chitosan*	–	1	6

* Reaction conditions: benzylamine (0.050 mol), solvent (30 mL), or without solvent, catalyst (1, 2, or 3 mol% based on Mn), 80 °C for 48 h in air. ** The relative standard deviation for all entries was below 5%, n = 5.

**Table 5:** Catalytic activity of Chitosan–Mn^2+^ systems in aldol reaction *.

Entry	Catalyst	Solvent	Conversion of 4-Nitrobenzaldehyde into the Aldol, % **			
			5 min	15 min	30 min	60 min
1	*Chitosan + Mn^2+^ (2:1)*	H_2_O	0	0	0	6
2	*Chitosan + Mn^2+^ (1:1)*	H_2_O	0	0	8	12
3	*Chitosan + Mn^2+^ (1:2*)	H_2_O	0	0	0	7
4	*Chitosan + Mn^2+^ (2:1)*	H_2_O/EtOH5/1 (*v*/*v*)	14	26	41	48
5	*Chitosan + Mn^2+^ (1:1)*	H_2_O/EtOH5/1 (*v*/*v*)	43	85	89	100
6	*Chitosan + Mn^2+^ (1:2*)	H_2_O/EtOH5/1 (*v*/*v*)	20	38	51	63
7	*–*	H_2_O/EtOH	0	0	0	0
8	*Mn^2+^*	5/1 (*v*/*v*)	0	0	0	0
9	*Chitosan*	H_2_O/EtOH	0	0	0	0
10	*Chitosan + Mn^2+^ (1:1)* *+ triethylbenzylammonium chloride (10%)*	H_2_O	9	20	38	45

* Reaction conditions: 4-nitrobenzaldehyde (0.010 mol), acetone (0.030 mol), catalyst (3 mol% based on Mn), and solvent (30 mL), 20 °C. ** The relative standard deviation for all entries was below 5%, n = 5.

## Data Availability

The original contributions presented in this study are included in the article. Further inquiries can be directed to the corresponding author.
